# An Inflammation-Related Nine-Gene Signature to Improve Prognosis Prediction of Lung Adenocarcinoma

**DOI:** 10.1155/2021/9568057

**Published:** 2021-09-18

**Authors:** Ze-jing Liu, Peng-xiao Hou, Xi-xing Wang

**Affiliations:** ^1^Department of Oncology, Shanxi Provincial Hospital of Traditional Chinese Medicine, Taiyuan City, Shanxi Province, China; ^2^Department of Traditional Chinese Medicine, Shanxi Cancer Hospital, Taiyuan City, Shanxi Province, China

## Abstract

**Background:**

A novel predictive model was rarely reported based on inflammation-related genes to explore clinical outcomes of lung adenocarcinoma (LUAD) patients.

**Methods:**

Using TCGA database, we screened nine inflammation-related genes with a prognostic value, and LASSO regression was applied for model construction. The predictive value of the prognostic signature developed from inflammation-related genes was assessed by survival assays and multivariate assays. PCA and t-SNE analysis were performed to demonstrate clustering abilities of risk scores.

**Results:**

Thirteen inflammation-related genes (BTG2, CCL20, CD69, DCBLD2, GPC3, IL7R, LAMP3, MMP14, NMUR1, PCDH7, PIK3R5, RNF144B, and TPBG) with prognostic values were finally identified. LASSO regression further screened nine candidates (BTG2, CCL20, CD69, IL7R, MMP14, NMUR1, PCDH7, RNF144B, and TPBG). Then, a prognostic prediction model using the above nine genes was constructed. A reliable clustering ability of risk score was demonstrated by PCA and t-SNE assays in 500 LUAD patients. The survival assays revealed that the overall survivals of the high-risk group were distinctly poorer than those of the low-risk group with 1-, 3-, and 5-year AUC values of 0.695, 0.666, and 0.694, respectively. Finally, multivariate assays demonstrated the scoring system as an independent prognostic factor for overall survival.

**Conclusions:**

Our study shows that the signature of nine inflammation-related genes can be used as a prognostic marker for LUAD.

## 1. Introduction

Lung cancer is the most common malignant tumor, constituting the leading cause of tumor-associated deaths worldwide [1]. It is classified into non-small-cell lung carcinoma (approximately 84% of cases) and small-cell lung carcinoma (approximately 16% of cases) [2]. Lung adenocarcinoma (LUAD) is the most common histological subtype of NSCLC [3]. Despite the fact that remarkable progresses in clinical treatments, such as neoadjuvant chemotherapy and surgery, have greatly improved the patients' survival rates, there still exist numerous patients suffering from distant metastasis [4, 5]. Therefore, there is an urgent need to develop a novel approach guiding clinical treatments and enhance the clinical outcome of LUAD patients.

Previous studies showed that the inflammatory microenvironment as the seventh hallmark of tumors could be activated to enhance tumor progression [6, 7]. LUAD has been reported to be associated with chronic bowel inflammation, indicating the crucial roles of inflammatory genes in the tumorigenesis and developments of LUAD [8, 9]. Additionally, a number of studies have reported the importance of single inflammatory genes in LUAD [10, 11]. For instance, the inflammation-related gene BTG2 was found to be lowly expressed in lung cancer and its overexpression suppressed the proliferation and metastasis of LUAD cells [12]. Besides, its diagnostic and prognostic value in lung cancer was also demonstrated in a previous study [13]. PCDH7 was shown to be distinctly overexpressed in LUAD, and its upregulation in cancers predicted a shorter survival of LUAD patients. Functionally, PCDH7 silence inhibited ERK activation and tumor growths [14]. Zhao and his group observed that three inflammatory genes (CSF3, IL-1A, and IL-6) were associated with long-term survivals of patients with B-cell lymphoma [15]. To date, there are no researches regarding a prevailing model based on inflammation-related genes for the prediction of clinical survivals of LUAD patients.

In this study, we aimed to define a prognostic inflammation-related gene signature capable of predicting overall survival in LUAD patients. A large cohort of patients with primary LUAD specimens and normal lung specimens from TCGA datasets were employed to screen differentially expressed inflammation-related genes. We screened inflammation-related genes that are distinctly associated with the outcome of LUAD, constructed a nine-mRNA model by the use of these genes, and delved into the prognostic values of the novel model in LUAD patients.

## 2. Materials and Methods

### 2.1. Microarray Datasets

Gene expression profile analysis data were obtained from TCGA datasets (https://portal.gdc.cancer.gov/). The data of LUAD tissues were used in the present study. The microarray data included 522 cases of LUAD. For the survival assays, 500 cases of LUAD including survival data were collected. Inflammation-related genes were extracted from the Molecular Signatures Database [16]. EdgeR-3.30.0 software was applied to analyze the differentially expressed genes (DEGs). By the use of the Benjamini and Hochberg (BH) methods, the corrected *p* value was obtained for the false discovery rate (FDR). mRNAs with FDR < 0.01, fold change > 2, and median of trans per million (TPM) > 5 were defined as having statistically significant differential expression. According to the National Center for Biotechnology Information database (https://www.ncbi.nlm.nih.gov), genes corresponding to these mRNAs were identified.

### 2.2. Clinical LUAD Sample Collection

A total of 8 paired primary LUAD tissues and corresponding nontumor tissues were collected from LUAD patients undergoing surgery at Shanxi Provincial Hospital of Traditional Chinese Medicine. The histopathological diagnosis of all samples was, respectively, diagnosed by two pathologists. Informed consent was obtained from all the patients. All experimental protocols were approved by the Institutional Review Committee of the Shanxi Provincial Hospital of Traditional Chinese Medicine.

### 2.3. Construction of the Prognostic Inflammation-Related Gene Signature for LUAD

After the prognostic inflammation-related genes of LUAD were screened with a *p* value of <0.01, Cox regression assays (using the “survival” package) were applied for the development of a prognostic model. According to initial analysis (*p* < 0.05), the collected inflammation-related genes were then incorporated into a least absolute shrinkage and selection operator- (LASSO-) penalized Cox proportional hazard regression model which was applied to recognize an optimal risk signature model without the risks of overfitting [17]. The model was applied to delve into the association between overall survival (OS) and inflammation-related genes. Then, our group used the model to calculate risk scores which were further applied to divide all patients into the high- and low-risk groups.

### 2.4. Assessment of Risk Score System

To explore the prognostic value of our model, Kaplan-Meier assays were carried out via the “survival” and “survminer” packages. Subsequently, the “survival ROC” package was applied to generate the receiver operating characteristic (ROC) curve. PCA and t-SNE assays were further conducted to assess the clustering ability of risk scores which can further demonstrate the relevancy of the model [18]. Univariate and multivariate assays were also conducted.

### 2.5. Quantitative Real-Time PCR Analysis

Total RNA was isolated from all tumor and normal specimens using Trizol reagent (Invitrogen). cDNA synthesis was performed with 2 mg of total RNA, using the miScript II RT Kit (Qiagen) according to the manufacturer's instructions. qRT-PCR assays were carried out by a protocol from Power SYBR Green (Takara, Hangzhou, Zhejiang, China). The relative expressions of genes were calculated and normalized using the 2^−*ΔΔ*Ct^ methods relative to GAPDH. Specific primer sequences are shown in Table 1.

### 2.6. Human Protein Atlas Analysis

The human protein at las (HPA; https://www.Proteinatlas.org/) comprises an atlas of human protein expression patterns in tumor and normal specimens. In this study, we examined the protein expressions of BTG2, MMP14, and PCDH7 using the HPA database.

### 2.7. Statistical Analysis

All analyses were conducted using R version 3.6.2. Differences were considered statistically significant at *p* < 0.05.

## 3. Results

### 3.1. The Identification of Prognostic Inflammation-Related Genes in LUAD

**Figure 1:**
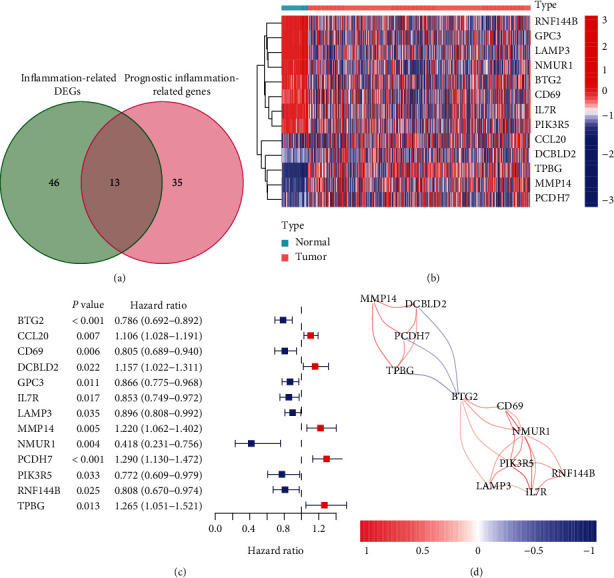
The identification of differentially expressed inflammation-related genes. (a) The prognostic inflammation-related genes associated with LUAD were demonstrated applying Venn diagram to study the intersection between inflammation-related genes and prognostic inflammation-related genes datasets. (b) Heatmap analysis of the prognostic DE inflammation-related genes. Microarray data were obtained from TCGA datasets. (c) Independent predictive power of the gene signature in LUAD patients.

Firstly, we analyzed TCGA datasets using “R” and screened 46 inflammation-related DEGs and 35 prognostic inflammation-related genes. Venn diagram showed 13 prognostic inflammation-related DEGs, including BTG2, CCL20, CD69, DCBLD2, GPC3, IL7R, LAMP3, MMP14, NMUR1, PCDH7, PIK3R5, RNF144B, and TPBG (Figure 1(a)). The heatmaps showed the expressed trends of the inflammation-related genes (Figure 1(b)). Univariate assays were conducted on 13 differential genes in 522 LUAD samples, and the *p* value and HR value are shown in Figure 1(c). Besides, we structured the correlation network based on the expression of BTG2, CCL20, CD69, DCBLD2, GPC3, IL7R, LAMP3, MMP14, NMUR1, PCDH7, PIK3R5, RNF144B, and TPBG in TCGA datasets and found that MMP14, DCBLD2, TPBG, and PCDH7 displayed a positive association. In addition, BTG2 was negatively associated with DCBLD2, PCDH7, and TPBG. BTG2, CD69, NMUR1, PIK3R5, LAMP3, RNF144B, and IL7R exhibited a positive association (Figure 1(d)).

### 3.2. Construction of Prognostic Inflammation-Related Gene Signature

**Figure 2:**
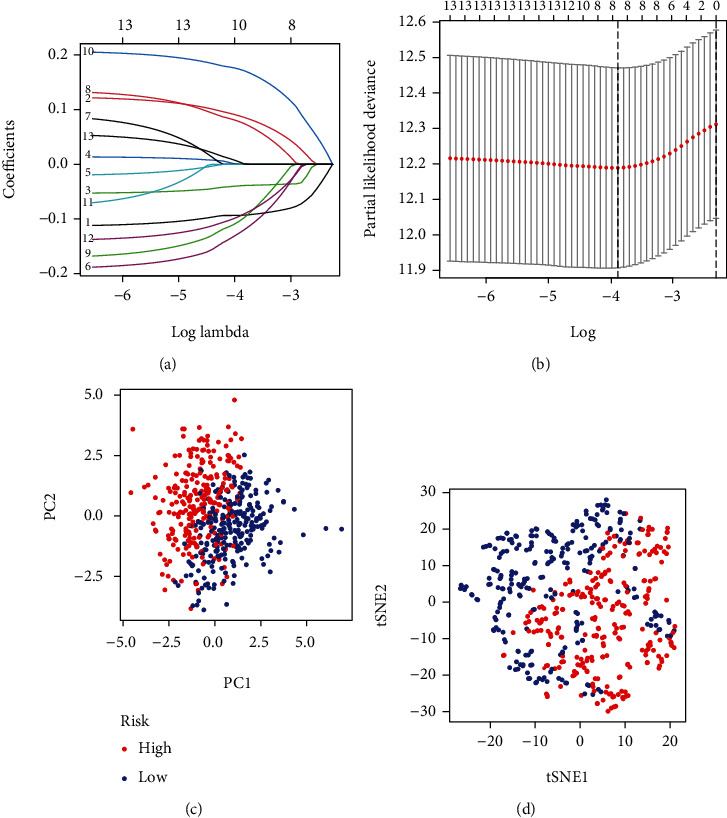
Construction of integrated risk score based on prognostic inflammation-related genes. (a, b) The LASSO Cox regression model was applied to construct the risk score system. (c) PCA and (d) t-SNE analysis.

To lower the risk of model overfitting, a LASSO regression of the above 13 genes was performed, resulting in the demonstration of 9 critical survival-associated inflammation-associated genes (Figures 2(a) and 2(b)). The nine genes were applied to establish the prognostic model score: risk score = (−0.0931193956074735 × BTG2) + (0.0858763560294805 × CCL20) + (−0.0389044638278403 × CD69) + (−0.120238398124069 × IL7R) + (0.0747331436403011 × MMP14) + (−0.0981341366623603 × NMUR1) + (0.171241040605377 × PCDH7) + (0.0911209619139391 × RNF144B) + (0.000901978433373243 × TPBG) (Table 2). Two of these 4 DE inflammation-related genes were associated with elevated risks (CCL20, MMP14, PCDH7, and TPBG; Coef > 0), whereas 5 were protective genes predicting decreased risks (BTG2, CD69, IL7R, NMUR1, and RNF144B; Coef < 0). All patients were scored by the use of this risk scoring methodology. All patients were separated into the low-risk (*n* = 250) and high-risk (*n* = 250) groups applying median risk score values.  Table 3 lists the clinical information of 250 LUAD patients. Moreover, PCA and t-SNE assays demonstrated the clustering abilities of this nine-gene-based risk score (Figures 2(c) and 2(d)).

### 3.3. The Nine-mRNA Model Had Strongly Diagnostic Power in the Prognostic Prediction

**Figure 3:**
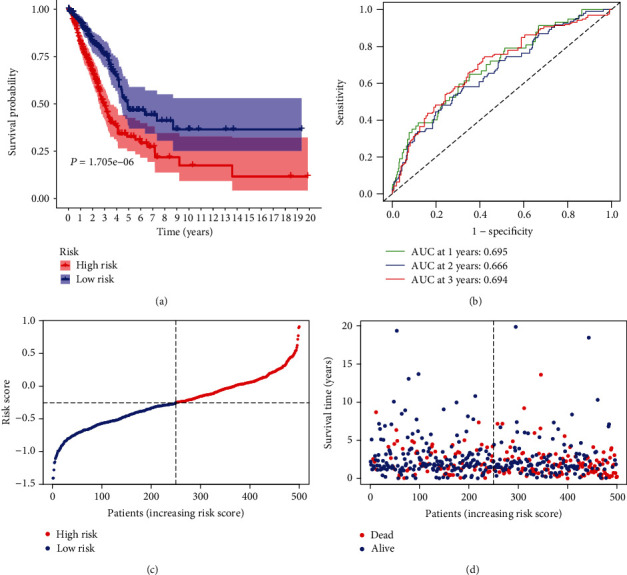
The prognostic value of the inflammation-related prognostic model for LUAD. (a) The survival assays between high-risk and low-risk groups. (b) ROC assays of the identified immune-related risk signature in 500 LUAD patients. (c) The risk score distribution of LUAD patients in 500 LUAD patients. (d) Survival status and survival time of each LUAD patient.

**Figure 4:**
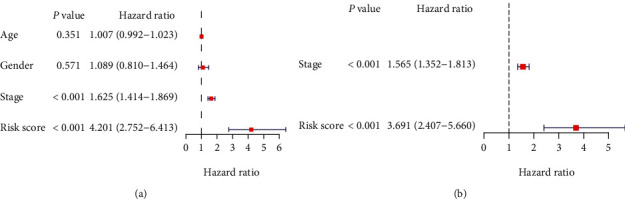
The nine inflammation-related genes' signature is an independent prognostic factor for LUAD. (a) Univariate Cox analysis showed that risk score (*p* < 0.001, HR = 4.201, 95% CI: 2.752-6.413) was correlated with OS of LUAD patients. (b) Multivariate Cox analysis demonstrated risk score (*p* < 0.001, HR = 3.691, 95% CI: 2.407-5.660) was independently associated with OS of LUAD patients.

Survival assays revealed that the overall survivals of patients in the high-risk group were distinctly shorter than those of patients in the low-risk group (*p* = 1.705*e* − 6; Figure 3(a)), with 1-, 3-, and 5-year AUC values of 0.695, 0.666, and 0.694, respectively (Figure 3(b)). The risk score distribution of LUAD patients in the TCGA datasets was shown (Figure 3(c)). A survival status overview was established (Figure 3(d)). Univariate assays revealed that stage (*p* < 0.001) and risk score (*p* < 0.001) could predict the OS of LUAD patients (Figure 4(a)). Multivariate assays further demonstrated that stage (*p* < 0.001) and the risk score (*p* < 0.001) could be independent biomarkers for LUAD patients (Figure 4(b)).

### 3.4. Data Validation

**Figure 5:**
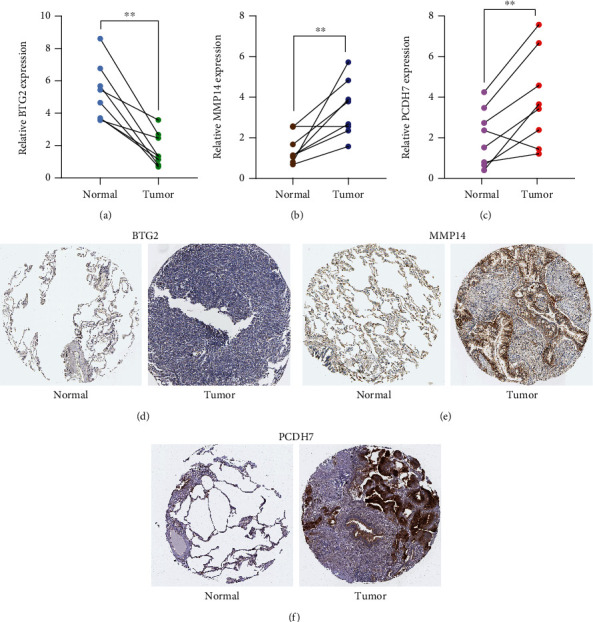
The expression of BTG2, MMP14 and PCDH7 in LUAD specimens. (a) BTG2. (b) MMP14. (c) PCDH7. (d–f) BTG2, MMP14, and PCDH7 expression in LUAD specimens and normal specimens from the Human Protein Atlas (HPA) database. ^∗∗^*p* < 0.01.

Then, we performed RT-PCR to examine the expression of BTG2, MMP14, and PCDH7 in LUAD specimens and observed that BTG2 expression (Figure 5(a)) was distinctly increased in normal lung specimens compared with normal lung specimens, while MMP14 (Figure 5(b)) and PCDH7 (Figure 5(c)) expressions were distinctly increased in LUAD specimens compared with normal lung specimens. Furthermore, immunohistochemistry data extracted from the HPA indicated that the protein expressions of BTG2 were higher in nontumor tissues compared with tumor specimens, while the expression of MMP14 and PCDH7 was lower in nontumor tissues compared with tumor specimens (Figures 5(d)–5(f)).

## 4. Discussion

The clinical treatments of LUAD remain a challenge, and LUAD is still the leading cause of tumor-associated mortality [19]. Although surgical resection is widely used, the 5-year survival rates are still about 15%, which shows that there is no satisfactory improvement in this area [2, 20]. In order to improve the clinical outcomes of LUAD patients, many researchers focused on the development of early diagnosis [21, 22]. Besides, more and more targeted therapies have been used to add therapeutic schedules in clinical practice, which proposed a higher demand for the identification of sensitive prognostic biomarkers [23, 24]. In recent years, more and more studies have revealed inflammatory genes as novel biomarkers due to their frequent dysregulation in both serum and tumor specimens as well as their oncogenic or antioncogenic roles in various tumors, including LUAD [25, 26].

In this study, we analyzed TCGA datasets and screen nine prognosis-related inflammatory genes (BTG2, CCL20, CD69, IL7R, MMP14, NMUR1, PCDH7, RNF144B, and TPBG), some of which were also demonstrated to exhibit a dysregulated expression in LUAD [27–29]. Previously, several above genes have been functionally studied in LUAD. For instance, BTG2 was highly expressed in lung cancer and promoted the proliferation and metastasis of tumor cells [30]. Overexpression of CCL20 promoted the induction of the lung cancer cell migration and proliferation through PI3K pathway [28]. MMP14 was also demonstrated to serve as a tumor promotor in lung cancer [29]. Those findings highlighted the potential of these inflammatory genes used as novel biomarkers. Thus, we performed multivariate assays and constructed the prognostic model which provided the risk score. By the use of the survival assays, risk assays, ROC curve, and multivariate assays, the accuracy of the model was further demonstrated. Moreover, we examined the expression of BTG2, MMP14, and PCDH7 in LUAD specimens and observed that BTG2 was lowly expressed in LUAD specimens, while MMP14 and PCDH7 were highly expressed in LUAD specimens. Thus, the signature was an independent predictive factor for LUAD patients.

Several limitations of our research should be noted. Firstly, the small number of patients were analyzed in this study; further studies on more patients are required to demonstrate our findings. Secondly, the potential function of the nine genes was not explored. Thus, more samples were necessary to demonstrate the accuracy of the prognostic model. Besides, more experiments are needed to elucidate the potential mechanisms involved in inflammation-related genes in LUAD progression.

## 5. Conclusion

Our study shows that the signature of nine inflammation-related genes can be used as a prognostic marker for LUAD.

## Figures and Tables

**Table 1:** Primers designed for qRT-PCR.

Name	Bidirectional primer sequence
BTG2:F	ACCACTGGTTTCCCGAAAAG
BTG2:R	CTGGCTGAGTCCGATCTGG
MMP14:F	GGCTACAGCAATATGGCTACC
MMP14:R	GATGGCCGCTGAGAGTGAC
PCDH7:F	GGATCGGGTGAGGTGACTTTC
PCDH7:R	GTTCTCGTCGAAGATCATCTGAC
GAPDH:F	ACAACTTTGGTATCGTGGAAGG
GAPDH:R	GCCATCACGCCACAGTTTC

**Table 2:** Nine genes associated with LUAD patient overall survivals.

Gene	Coef
BTG2	-0.0931193956074735
CCL20	0.0858763560294805
CD69	-0.0389044638278403
IL7R	-0.120238398124069
MMP14	0.0747331436403011
NMUR1	-0.0981341366623603
PCDH7	0.171241040605377
RNF144B	-0.0911209619139391
TPBG	0.000901978433373243

**Table 3:** Clinical characteristics of patients with LUAD in different risk groups.

Parameters	Group	Total (500)	High risk (250)	Low risk (250)	*p* value
Gender	Male	230	125	105	0.073
	Female	270	125	145	

Age (years)	<65	219	127	92	0.002
	≥65	281	123	158	

Clinical stage	I-II	387	183	204	0.023
	III-IV	113	67	46	

## Data Availability

The data included in the current study were available in TCGA database (https://cancergenome.nih.gov/).
